# Regional Transcriptomic Architecture of Glioblastoma Reveals NUCB2 as a Key Orchestrator of Tumour Aggression and Immune Dysfunction

**DOI:** 10.1111/jcmm.70814

**Published:** 2025-09-06

**Authors:** Xiaodong Huang, Xuemei Hu, Hang Xu, Bowen Yang, Lidong Yan, Junti Lu, Kuanming Huang, Huibin Chen, Shengli Hu

**Affiliations:** ^1^ Department of Neurosurgery Taihe Hospital, Hubei University of Medicine Shiyan City Hubei Province China; ^2^ Department of Nephrology Taihe Hospital, Hubei University of Medicine Shiyan City Hubei Province China; ^3^ Department of Critical Care Medicine Taihe Hospital, Hubei University of Medicine Shiyan City Hubei Province China

**Keywords:** glioblastoma, immune dysfunction, NUCB2, tumour microenvironment

## Abstract

Glioblastoma (GBM) exhibits remarkable intra‐tumoral heterogeneity, which contributes to therapeutic resistance and poor clinical outcomes. In this study, we employed integrative single‐cell RNA sequencing analysis across two complementary public datasets encompassing diverse cellular populations from GBM centre and periphery regions to elucidate potential spatial molecular programmes driving tumour progression. Our analyses revealed substantial transcriptomic divergence between anatomically distinct tumour regions, with NUCB2 emerging as significantly upregulated in centre‐residing neural progenitor cell‐like (NPC‐like) tumour cells. Functional characterisation demonstrated NUCB2's critical role in regulating GBM stem cell proliferation, with knockdown experiments resulting in significant reduction in tumour cell growth. Intriguingly, NUCB2 expression strongly associated with immunosuppressive molecular signatures and paradoxical immune cell infiltration patterns. Specifically, CD8+ T cells from GBM centre regions exhibited distinctive transcriptional programmes enriched for interferon response, complement activation, and inflammatory pathways, suggesting a state of functional impairment despite enhanced infiltration. Survival analyses confirmed that elevated NUCB2 expression significantly associated with poorer patient survival. Collectively, our findings establish NUCB2 as a multifaceted regulator that coordinates both intrinsic proliferative capacity and extrinsic immunomodulatory functions within the GBM microenvironment. This previously uncharacterised NUCB2‐driven axis represents a promising therapeutic target, potentially enabling simultaneous targeting of tumour cell proliferation and immune evasion mechanisms in this aggressive malignancy.

## 1 Introduction

Glioblastoma (GBM) is one of the most lethal primary brain malignancies, with a dismal median survival duration of approximately 15 months despite the application of intensive multimodal therapy [1, 2]. This therapeutic resistance stems largely from the pronounced heterogeneity of GBM, which includes a complex array of diverse cellular populations, molecular profiles and microenvironmental interactions that collectively drive tumour progression and confer treatment resistance [3, 4]. Additionally, the complicated spatial architecture of GBM introduces an additional layer of complexity, as distinct molecular pathways are differentially active within the central tumour regions versus the infiltrative periphery regions [5].

Recent technological advances in single‐cell RNA sequencing (scRNA‐seq) have significantly enhanced our understanding of the cellular ecosystems within GBM, revealing previously unrecognised heterogeneity in both neoplastic and non‐neoplastic regions [6, 7, 8]. These high‐resolution molecular characterisations have illuminated the complex and dynamic interplays between tumour cells and their nearby microenvironment, particularly the immune landscape, which exhibits paradoxical behaviour in GBM relative to other solid malignancies [9, 10, 11]. While increased immune infiltration generally associates with improved outcomes across multiple cancer types, GBM often demonstrates the inverse correlation, indicating fundamental alterations in immune cell functions within the unique confines of the brain tumour microenvironment [12, 13, 14].

Nucleobindin‐2 (NUCB2), a multifunctional calcium‐binding protein originally characterised for its roles in hypothalamic signalling and metabolic regulation [15, 16, 17, 18], has recently been implicated as a potential mediator in multiple malignancies [19, 20, 21]. However, its specific functions within GBM, particularly concerning spatial distribution and immunomodulatory capabilities, remain insufficiently characterised. Previous studies have suggested that the role of NUCB2 in cellular proliferation and stress responses in different cancers; however, a comprehensive functional understanding in the context of brain malignancies is notably lacking currently.

In this study, we leverage integrative analyses of single‐cell transcriptomic datasets from complementary GBM samples to dissect the spatial molecular heterogeneity between tumour core and peripheral regions. Our findings uncover NUCB2 as an important differentially expressed gene (DEG) in central NPC‐like tumour cells and reveal its dual functionality in both promoting tumour cell proliferation and modulating the local immune microenvironment. Through functional validation and correlative analyses, we reveal a previously unrecognised function of NUCB2 in driving GBM aggressiveness through parallel regulation of intrinsic tumour growth and extrinsic immune evasion mechanisms. These findings not only expand our fundamental understanding of GBM pathobiology but also identify NUCB2 as a promising therapeutic target with the potential to address multiple hallmarks of devastating malignancy.

## 2 Results

### 2.1 Spatial Variability of Immune Cell Populations Within Glioblastoma Microenvironments

**FIGURE 1:**
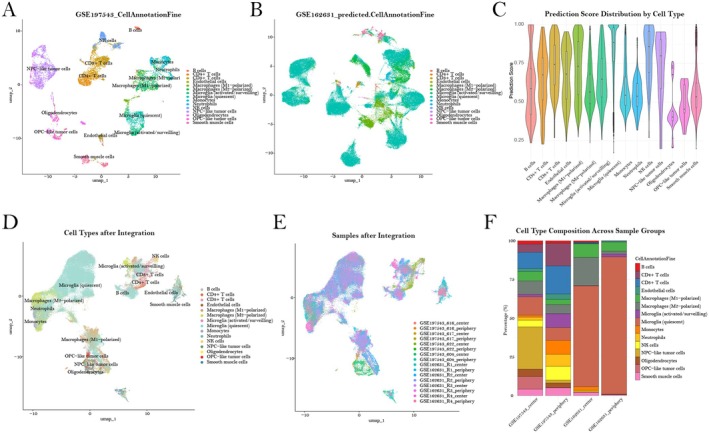
Integration and comparative characterisation of immune cell populations within glioblastoma microenvironments. (A) UMAP visualisation of cell type distribution in the GSE197543 dataset, showing distinct clustering patterns of diverse cell populations derived from GBM central and peripheral regions. (B) UMAP visualisation of the GSE162631 dataset following cell type annotation using Seurat label transfer method, highlighting CD31‐positive immune cells isolated from GBM central and peripheral tissues. (C) Violin plot displaying the distribution of prediction scores across identified cell types, with the majority exceeding 0.6, thereby supporting the robustness of the label transfer approach. (D) UMAP visualisation post‐integration of datasets, demonstrating improved clustering coherence among immune cell populations. (E) UMAP plot depicting sample distribution subsequent to integration, showing balanced mixing of samples within the projection space. (F) Stacked bar chart presenting differential cell type composition between GBM central and peripheral regions across sample groups, highlighting significant variations in immune cell proportions.

Notably, immune cell heterogeneity within glioblastoma microenvironments exhibits quite profound spatial variation, which may potentially influence tumour progression in critical ways. To get a thorough picture of this complex heterogeneity, we analysed two complementary single‐cell RNA sequencing datasets: GSE197543 [22], encompassing diverse cell populations from GBM centre and periphery regions (Figure 1A), and GSE162631 [23], comprising CD31+ immune cells that were specifically isolated from the corresponding regions (Figure 1B). Initial cell type annotation was performed via Seurat label transfer method, with prediction scores predominantly above 0.6 across virtually all identified cell types (Figure 1C), thereby substantiating the overall fidelity of our annotation approach. Despite the somewhat diffuse clustering patterns that were observed in the GSE162631 dataset, integration of both datasets using canonical correlation analysis appeared to significantly enhance clustering resolution (Figure 1D) while still maintaining balanced representation across sample origins (Figure 1E). Importantly, this integration strategy not only mitigated batch effects but also augmented immune cell representation, thereby potentially bolstering analytical robustness. Interestingly, quantitative assessment of cellular compositions revealed striking disparities in immune cell proportions between GBM centre and periphery microenvironments (Figure 1F). These distinct immune landscapes imply the existence of region‐specific immunomodulatory mechanisms that may differentially influence various aspects of GBM pathophysiology and progression.

### 2.2 Transcriptomic Variations in NPC‐Like Tumour Cells Between Centre and Peripheral Regions of Glioblastoma

**FIGURE 2:**
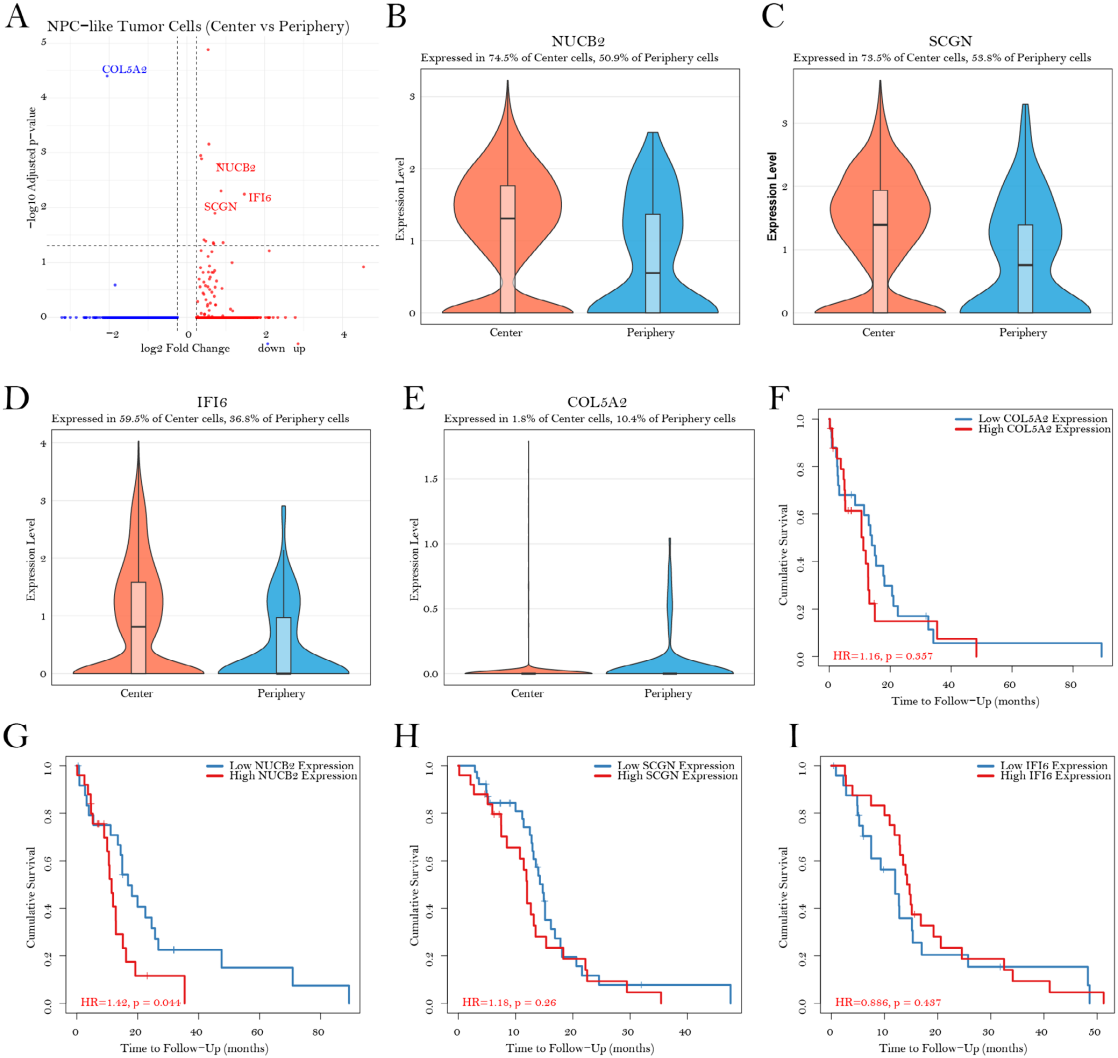
Differential gene expression profiling of NPC‐like tumour cells in GBM central versus peripheral regions and associated prognostic significance. (A) Volcano plot illustrates differentially expressed genes in NPC‐like tumour cells comparing central versus peripheral regions. Blue dots represent significantly downregulated genes, while red dots indicate significantly upregulated genes in central regions (adjusted *p*‐value < 0.05, |log_2_ fold‐change| > 0.25). Key genes of interest (NUCB2, SCGN, IFI6 and COL5A2) are annotated. (B–E) Violin plots depicting expression distributions of selected differentially expressed genes: (B) NUCB2 (expressed in 74.5% of central cells vs. 50.9% of peripheral cells), (C) SCGN (73.5% vs. 53.8%), (D) IFI6 (59.5% vs. 36.8%) and (E) COL5A2 (1.8% vs. 10.4%). (F–I) Kaplan–Meier survival analyses examining the relationship between gene expression levels and patient outcomes for (F) COL5A2 (HR = 1.16, *p* = 0.357), (G) NUCB2 (HR = 1.42, *p* = 0.044), (H) SCGN (HR = 1.18, *p* = 0.26) and (I) IFI6 (HR = 0.886, *p* = 0.437). In these plots, red curves correspond to high expression groups, whereas blue curves represent low expression groups.

A comparative transcriptomic analysis of NPC‐like tumour cells isolated from centre and peripheral regions of glioblastoma multiforme uncovered distinct gene expression profiles associated with spatial localisation. Differential expression analysis identified several significantly dysregulated genes between these regions (adjusted *p* < 0.05, |log_2_ fold change| > 0.25). Specifically, NUCB2, SCGN and IFI6 were markedly upregulated in cells from the tumour centre, whereas COL5A2 was significantly downregulated in this region (Figure 2A). Detailed expression profiling demonstrated that NUCB2 was expressed in 74.5% of central tumour cells compared to only 50.9% in peripheral cells (Figure 2B). Comparable regional expression differences were observed for SCGN (73.5% vs. 53.8%, Figure 2C) and IFI6 (59.5% vs. 36.8%, Figure 2D). In contrast, COL5A2 expression was minimal in central cells (1.8%) but present in 10.4% of peripheral cells (Figure 2E). To assess the potential clinical significance of these spatially regulated genes, survival analyses were conducted using the TIMER 2.0 platform on The Cancer Genome Atlas (TCGA) GBM patient cohort (*n* = 153). Notably, elevated NUCB2 expression was significantly associated with reduced overall survival (hazard ratio [HR] = 1.42, *p* = 0.044, Figure 2G). Conversely, COL5A2 (HR = 1.16, *p* = 0.357, Figure 2F), SCGN (HR = 1.18, *p* = 0.26, Figure 2H) and IFI6 (HR = 0.886, *p* = 0.437, Figure 2I) demonstrated no statistically significant correlations with patient outcomes. These results suggest that NUCB2 may serve not only as a marker of spatial heterogeneity within GBM but also as a potential prognostic biomarker, highlighting the critical importance of region‐specific molecular characterisation in elucidating GBM pathobiology and progression dynamics.

### 2.3 Elevated Expression of NUCB2 in GBM and Its Critical Function in Tumour Cell Proliferation

**FIGURE 3:**
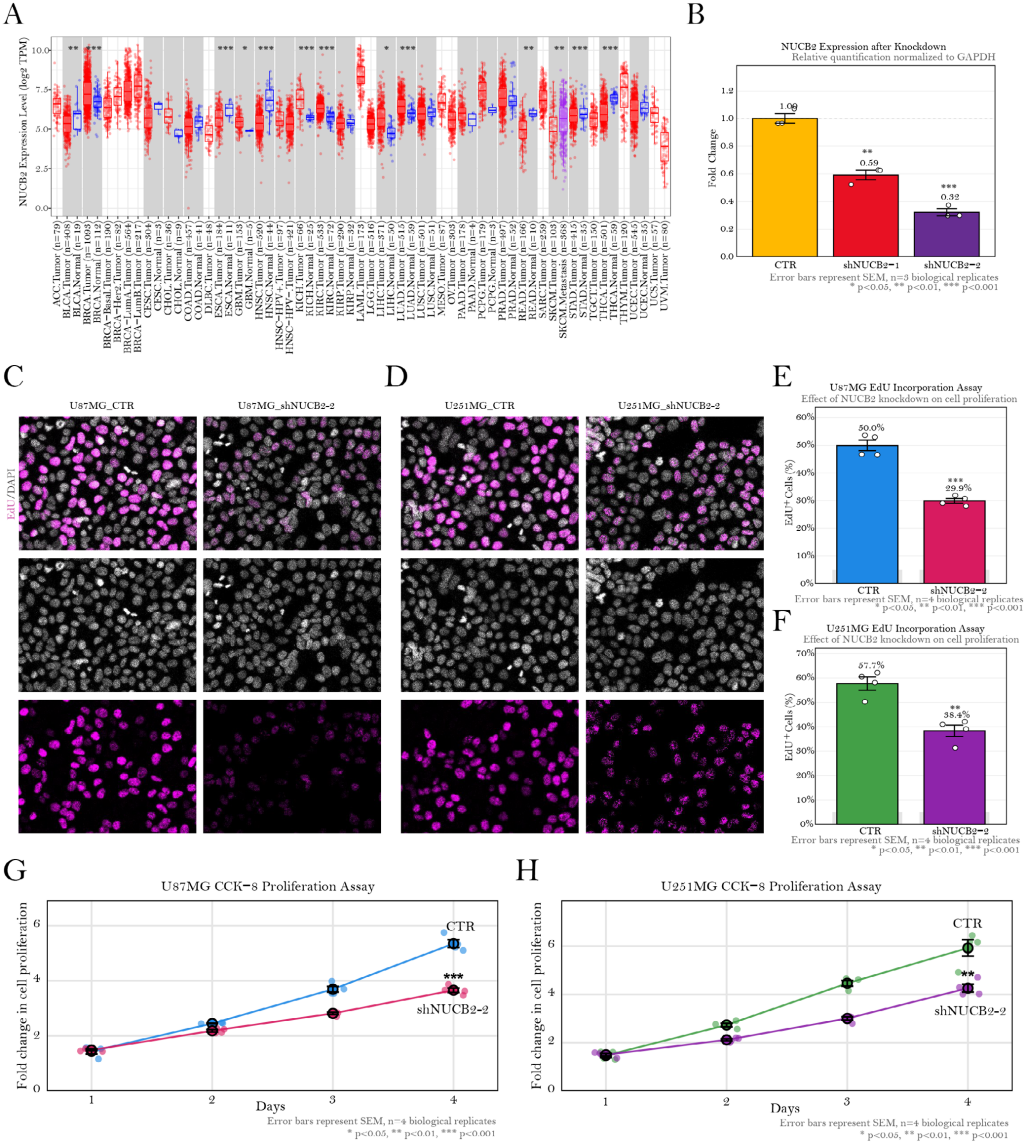
Expression of NUCB2 across various cancer types and its functional impact in GBM cell proliferation. (A) A pan‐cancer analysis of NUCB2 expression was conducted using TIMER 2.0, comparing tumour tissues (depicted in red) with corresponding normal tissues (depicted in blue) across multiple cancer types. NUCB2 is significantly upregulated in several cancer types including BRCA, ESCA, GBM, HNSC, KICH, LIHC, LUAD, READ, SKCM, STAD and THCA. (B) qPCR analysis assessed the knockdown efficiency of NUCB2 using two distinct shRNA constructs (shNUCB2‐1 and shNUCB2‐2) normalised to GAPDH expression. (C, D) Representative fluorescence microscopy images from EdU incorporation assays in (C) U87MG and (D) U251MG GBM cell lines under control and NUCB2 knockdown conditions are presented. The upper panels display merged images showing EdU (magenta) and DAPI (grey), the middle panels display the DAPI channel alone and the lower panels depict the EdU channel. (E, F) Quantitative analysis of EdU‐positive cells, expressed as a percentage of total cells, is shown for (E) U87MG and (F) U251MG cell lines. NUCB2 knockdown significantly decreased the proportion of proliferating cells in both U87MG and U251MG cell lines. (G, H) Cell Counting Kit‐8 (CCK‐8) proliferation assays over a 4‐day period were performed in (G) U87MG and (H) U251MG cells transfected with either control vector (CTR) or NUCB2‐targeting shRNA (shNUCB2). NUCB2 knockdown resulted in a progressive decline in proliferative capacity in both cell lines. Error bars represent the standard error of the mean (SEM) from four biological replicates. Statistical significance is indicated as follows: *p < 0.05, **p < 0.01, ***p < 0.001.

A comprehensive pan‐cancer analysis revealed that NUCB2 expression is markedly elevated in various cancer types compared to their corresponding normal tissues (Figure 3A). Of particular note, GBM exhibited significant upregulation of NUCB2, alongside other malignancies such as breast carcinoma (BRCA), oesophageal carcinoma (ESCA), head and neck squamous cell carcinoma (HNSC), kidney chromophobe carcinoma (KICH), liver hepatocellular carcinoma (LIHC), lung adenocarcinoma (LUAD), colorectal adenocarcinoma (READ), skin cutaneous melanoma (SKCM), stomach adenocarcinoma (STAD) and thyroid carcinoma (THCA). To characterise the functional role of NUCB2 in GBM, transient knockdown experiments were conducted using two distinct short hairpin RNA (shRNA) constructs targeting NUCB2. Quantitative PCR analysis confirmed effective suppression of NUCB2 expression with both constructs, with reductions of 41% (shNUCB2‐1, *p* < 0.01) and 68% (shNUCB2‐2, *p* < 0.001) compared to control cells (Figure 3B). Given the superior knockdown efficiency, shNUCB2‐2 was selected for subsequent functional assays. EdU incorporation assays performed in U87MG and U251MG GBM cell lines revealed a significant impairment in cellular proliferation following NUCB2 depletion (Figure 3C,D). Quantitative assessment revealed a substantial reduction in the proportion of EdU‐positive proliferating cells in both U87MG (29.9% vs. 50.0% in control, *p* < 0.001; Figure 3E) and U251MG (38.4% vs. 57.7% in control, *p* < 0.01; Figure 3F) cell lines upon NUCB2 knockdown. To further confirm the effect of NUCB2 depletion on cellular proliferation, we performed CCK‐8 time‐course assays using the U87MG and U251MG cell lines. In alignment with the EdU incorporation data, knockdown of NUCB2 significantly reduced the proliferative capacity in both cell lines (Figure 3G,H). Collectively, these longitudinal data corroborate the EdU assay results and underscore the pivotal role of NUCB2 in maintaining the enhanced proliferative capacity characteristic of glioblastoma cells. Collectively, these findings identify NUCB2 as a critical regulator of GBM cell proliferation, corroborating its elevated expression in tumour tissues and underscoring its potential as a therapeutic target in GBM.

### 2.4 NUCB2 Expression Correlates With an Immunosuppressive Tumour Microenvironment and Adverse Immune Cell Infiltration in GBM

**FIGURE 4:**
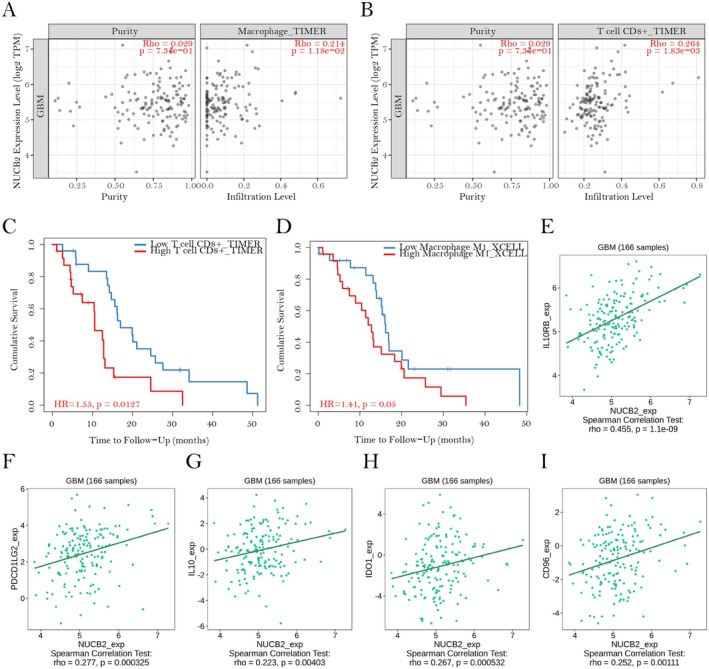
Association between NUCB2 expression, immune cell infiltration and immunoinhibitory molecules in GBM. (A, B) Correlation analyses between NUCB2 expression and immune cell infiltration in GBM were conducted utilising TIMER 2.0. Specifically, (A) macrophage infiltration exhibited a positive correlation with NUCB2 expression (Spearman's *ρ* = 0.214, *p* = 1.18 × 10^−2^) and (B) CD8+ T‐cell infiltration also correlated positively (*ρ* = 0.264, *p* = 1.83 × 10^−3^). The left panels include adjustments for tumour purity. (C, D) Kaplan–Meier survival analyses stratified GBM patients based on infiltration levels of (C) CD8+ T cells (hazard ratio [HR] = 1.55, *p* = 0.0127) and (D) M1 macrophages (HR = 1.41, *p* = 0.05). High and low infiltration groups are represented by red and blue lines, respectively. (E–I) Correlation analyses between NUCB2 expression and immunoinhibitory molecules in GBM samples (*n* = 166) from the TISIDB database revealed significant positive associations with (E) IL10RB (*ρ* = 0.455, *p* = 1.1 × 10^−9^), (F) PDCD1LG2 (*ρ* = 0.277, *p* = 3.25 × 10^−4^), (G) IL10 (*ρ* = 0.223, *p* = 4.03 × 10^−3^), (H) IDO1 (*ρ* = 0.267, *p* = 5.32 × 10^−4^) and (I) CD96 (*ρ* = 0.252, *p* = 1.11 × 10^−3^). Linear regression fits are depicted by green lines.

To investigate the potential immunological roles of NUCB2 in GBM, we examined its association with immune cell infiltration and immunoregulatory factors. Analysis using TIMER 2.0 revealed significant positive correlations between NUCB2 expression and the infiltration levels of macrophages (Spearman's *ρ* = 0.214, *p* = 1.18e‐02; Figure 4A) as well as CD8+ T cells (Spearman's *ρ* = 0.264, *p* = 1.83e‐03; Figure 4B) within GBM tissues. Strikingly, survival analyses indicated that elevated infiltration of both CD8+ T cells (HR = 1.55, *p* = 0.0127; Figure 4C) and M1 macrophages (HR = 1.41, *p* = 0.05; Figure 4D) was correlated with poorer clinical outcomes in GBM patients, a finding that contrasts with their conventionally anti‐tumoral roles in various cancer types. This apparent paradox prompted us to further examine whether NUCB2 might contribute to an immunosuppressive tumour microenvironment. Consistently, TISIDB analysis revealed robust positive correlations between NUCB2 expression and several immunoinhibitory molecules in GBM samples (*n* = 166), including IL10RB (*ρ* = 0.455, *p* = 1.1e‐09; Figure 4E), PDCD1LG2 (*ρ* = 0.277, *p* = 0.000325; Figure 4F), IL10 (*ρ* = 0.223, *p* = 0.00403; Figure 4G), IDO1 (*ρ* = 0.267, *p* = 0.000532; Figure 4H) and CD96 (*ρ* = 0.252, *p* = 0.00111; Figure 4I). Collectively, these results suggest that NUCB2 may play a critical role in establishing an immunosuppressive tumour microenvironment in GBM, potentially impairing the functionality of infiltrating immune cells or even promoting tumour‐supportive phenotypes, thereby contributing to immune evasion and aggressive clinical progression characteristic of this malignancy.

### 2.5 Distinct Transcriptional Profiles of CD8+ T Cell in GBM Microenvironments Correlate With NUCB2‐Driven Immunosuppressive Mechanisms

**FIGURE 5:**
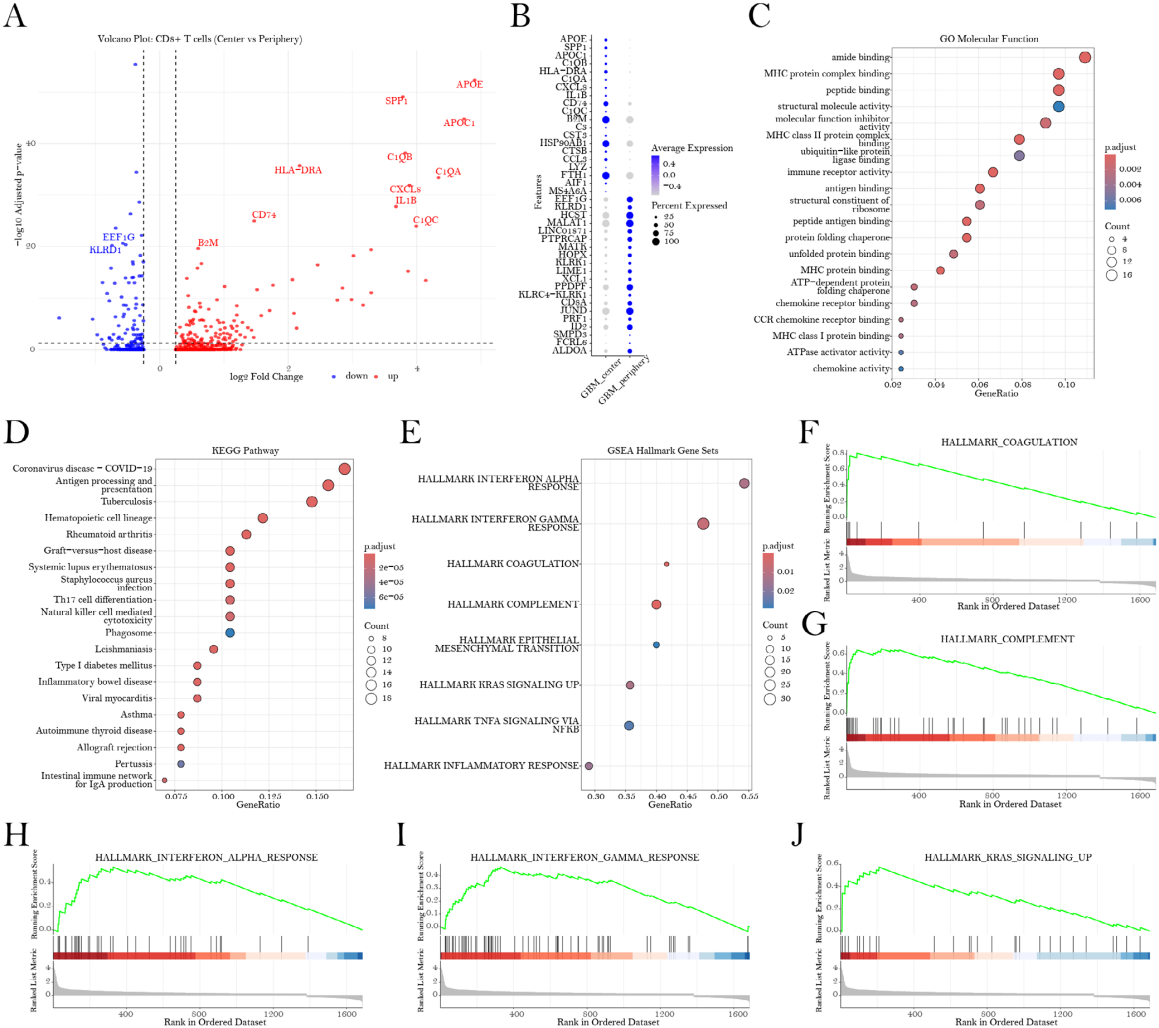
Transcriptomic characterisation and functional enrichment analysis of CD8+ T cells isolated from the central versus peripheral regions of GBM. (A) Volcano plot illustrates the differentially expressed genes in CD8+ T cells when comparing central versus peripheral regions. Red dots indicate significantly upregulated genes (APOE, APOC1, HLA‐DRA, C1QA, C1QB, C1QC, IL1B, CCL3, CD74) in central regions, while blue dots (KLRD1, FLT3LG) represent significantly downregulated genes (adjusted *p*‐value < 0.05, |log_2_ fold‐change| > 0.25). (B) Dot plot presenting the expression profiles of the top 40 differentially expressed genes, including 20 upregulated and 20 downregulated genes between central and peripheral CD8+ T cells. The size of each dot corresponds to the proportion of cells expressing the gene, while the colour intensity reflects the average expression level. (C) Gene Ontology (GO) molecular function enrichment analysis of the differentially expressed genes reveals significant enrichment in functions related to MHC protein complex binding, peptide binding and immune receptor activity. (D) KEGG pathway analysis identifies significant enrichment in pathways associated with antigen processing and presentation, as well as T‐cell differentiation. (E) GSEA hallmark gene set enrichment analysis showing significant enrichment in pathways involved in interferon responses, coagulation, complement and inflammatory processes. (F–J) Enrichment plots for selected hallmark gene sets: (F) HALLMARK_COAGULATION, (G) HALLMARK_COMPLEMENT, (H) HALLMARK_INTERFERON_ALPHA_RESPONSE, (I) HALLMARK_INTERFERON_GAMMA_RESPONSE and (J) HALLMARK_KRAS_SIGNALING_UP.

A comparative transcriptomic investigation of CD8+ T cells isolated from the central versus peripheral regions of GBM samples revealed substantial gene expression differences that potentially explain the paradoxical association between elevated CD8+ T‐cell infiltration and adverse clinical patient outcomes. Differential gene expression analysis identified 254 genes exhibiting significant alterations (adjusted *p* < 0.05), with notable upregulation of immunoregulatory genes such as APOE and APOC1, major histocompatibility complex (MHC) class II components including HLA‐DRA, complement system factors (C1QA, C1QB, C1QC), and pro‐inflammatory mediators (IL1B, CCL3) in CD8+ T cells localised to the tumour core (Figure 5A,B). Functional enrichment analyses uncovered significant overrepresentation of molecular functions related to antigen binding and immune receptor activity (Figure 5C), while Kyoto Encyclopedia of Genes and Genomes (KEGG) pathway analysis underscored pathways involved in antigen processing and T‐cell differentiation (Figure 5D). Gene set enrichment analysis (GSEA) further revealed remarkable enrichment of interferon response signatures, complement activation, coagulation cascades and inflammatory pathways within central CD8+ T cells (Figure 5E–J). Collectively, these data suggest that CD8+ T cells residing in the GBM core adopt a complex and partially dysfunctional phenotype characterised by augmented interferon signalling and inflammatory activation, which may underline their unexpected association with poorer clinical outcomes. Importantly, the implicated pathways correspond with our prior observations concerning NUCB2‐related immunoregulatory molecules, suggesting a putative immunomodulatory axis wherein NUCB2 expression by tumour cells contributes to CD8+ T‐cell dysfunction via enhanced interferon signalling and complement activation. This mechanism likely promotes an immunosuppressive tumour microenvironment that facilitates disease progression despite increased infiltration of immune effector cells.

## 3 Discussion

Our extensive analysis of the spatial molecular heterogeneity of glioblastoma identifies NUCB2 as a critical regulator situated at the intersection of tumour cell proliferation and immune evasion. Utilising integrative analysis of public single‐cell transcriptomic datasets, we reveal that NUCB2 is markedly upregulated in NPC‐like tumour cells located within the central regions of GBM compared to peripheral areas, suggesting a region specific set of molecular programmes. This spatial variation in NUCB2 expression is correlated with distinct tumour microenvironmental characteristics that collectively contribute to the well‐documented therapeutic resistance observed in GBM.

The functional relevance of NUCB2 in GBM pathophysiology is evidenced by our knockdown experiments, which demonstrate a substantial reduction in cellular proliferation in both U87MG and U251MG cell lines. These findings corroborate emerging evidence implicating NUCB2 in proliferative mechanisms across various cancer types, including nasopharyngeal carcinoma, bladder cancer, hepatocellular carcinoma, colorectal cancer and thyroid cancer [24, 25, 26, 27, 28]. Importantly, our study extends beyond this established paradigm by revealing a novel immunomodulatory role of NUCB2 within the GBM ecosystem. Specifically, the positive association between NUCB2 expression and several immunoinhibitory molecules, such as IL10RB, PDCD1LG2, IL10, IDO1 and CD96, suggests that NUCB2 may facilitate the establishment of an immunosuppressive microenvironment, potentially accounting for the paradoxical observation that increased immune infiltration correlates with poorer clinical outcomes in GBM patients.

Of particular interest is our observation that CD8+ T cells derived from GBM central regions, characterised by elevated NUCB2 expression, exhibit unique transcriptional profiles enriched for interferon signalling, complement activation and inflammatory pathways. This pattern likely reflects a state of T‐cell dysfunction or exhaustion induced by the tumour microenvironment. Although the precise mechanistic relationship between tumour‐expressed NUCB2 and these alternations in T‐cell phenotype requires further investigation, our findings suggest a putative axis whereby NUCB2 promotes the production of immunomodulatory factors that subsequently modulate the functional state of infiltrating T cells.

A notable finding of this study is the clinical significance of NUCB2 expression, which exhibits a strong association with reduced overall survival rate in patients diagnosed with GBM. This prognostic relevance, combined with the involvement of NUCB2 in both tumour cell proliferation and immune regulation, underscores its potential as a promising therapeutic capable of eliciting multifaceted effects. Therapeutic strategies aimed at inhibiting NUCB2 may simultaneously suppress tumour growth and enhance anti‐tumour immune responses, representing a particularly compelling approach for addressing the therapeutic challenges posed by GBM.

The blood–brain barrier (BBB) constitutes an essential obstacle for the delivery of NUCB2‐targeted therapies. Adeno‐associated virus (AAV) vectors, especially serotype AAV9 and engineered variants such as AAV‐PHP.eB, have demonstrated efficient central nervous system (CNS) tropism and the ability to traverse the blood–brain barrier following systemic administration. These vectors offer a viable platform for delivering gene‐silencing agents, including shRNA or CRISPR‐Cas components directed against NUCB2. Alternative delivery modalities, such as nanoparticle systems exploiting transferrin receptor‐mediated transcytosis or employing cell‐penetrating peptides, also present promising pharmaceutical strategies. Furthermore, the intrinsic vascular permeability characteristic of GBM may facilitate the selective accumulation of suitably engineered therapeutics, thereby enabling targeted modulation of NUCB2 within the GBM tumour microenvironment. Tumour heterogeneity remains a critical challenge for the efficacy of NUCB2‐targeted interventions. Tumour regions enriched with NUCB2‐expressing NPC‐like populations may exhibit enhanced sensitivity to such targeted therapies, whereas areas dominated by NUCB2‐low cellular subsets could maintain their resistance to treatment. Addressing this intratumoral variability could be important for optimising the therapeutic potential of the inhibition of NUCB2 in GBM.

### 3.1 Limitations

Several limitations inherent to our present study warrant careful consideration. Firstly, although single‐cell RNA sequencing affords unparalleled resolution in characterising cellular heterogeneity, it inherently provides only a static snapshot of dynamic biological processes. Moreover, the mechanistic framework positing a link between NUCB2 expression and CD8+ T‐cell dysfunction, while compelling, remains an incompletely understood paradigm requiring further in‐depth exploration. Our transcriptomic analysis findings reveal seemingly paradoxical signatures characterised by elevated interferon responses and inflammatory activation coexisting with apparent functional impairment in CD8+ T cells. This apparent contradiction likely reflects complex immunoregulatory cascades whereby NUCB2‐expressing tumour cells secrete immunomodulatory factors that initially promote T‐cell activation but subsequently induce exhaustion phenotypes. Several plausible mechanisms merit consideration: NUCB2 may modulate the production of immunosuppressive cytokines or metabolites that remodel the local tumour microenvironment, thereby fostering conditions conducive to T‐cell dysfunction despite robust transcriptional activation. Alternatively, the observed enrichment of complement and coagulation pathways suggests additional layers of complexity. Critically, these correlative observations require rigorous functional validation using sophisticated in vivo models that preserve intact tumour–immune interactions. Only through such mechanistic studies can causality between the NUCB2 expression and the distinctive T‐cell phenotypes identified be definitively established.

Secondly, our use of shRNA‐mediated knockdown method, although yielding reproducible proliferative phenotypes across two GBM cell lines (U87MG and U251MG), inherently provides only transient suppression of NUCB2. Employing CRISPR‐Cas9‐mediated permanent genetic ablation would likely uncover phenomena obscured by the temporal limitations of transient knockdown, particularly those involving complex immune–tumour interactions evolving over extended periods. Sustained manipulation may reveal compensatory mechanisms, adaptive responses, or subtle perturbations within the tumour microenvironment that are not detectable through short‐term analyses. Additionally, the single‐cell transcriptomic approach employed captures static profiles rather than dynamic cellular processes. Future studies utilising stable knockout models in conjunction with advanced co‐culture systems or orthotopic tumour models are anticipated to yield unprecedented insights into the temporal regulatory dynamics of NUCB2.

Future research directions should focus on exploring the precise molecular mechanisms by which NUCB2 modulates both tumour cell‐intrinsic properties and the broader tumour microenvironment. Investigations into therapeutic strategies involving NUCB2, either as monotherapy or in combination with immune checkpoint inhibitors, represent a promising avenue. Furthermore, exploring the role of NUCB2 in GBM recurrence and therapeutic resistance may provide valuable insights into its potential utility as a biomarker for treatment stratification.

In summary, our study identifies NUCB2 as a multifaceted regulator in GBM, orchestrating both proliferative capacity and immunomodulatory functions that collectively contribute to tumour progression. These findings not only advance the fundamental understanding of GBM biology but also highlight NUCB2 as a promising therapeutic target capable of simultaneously addressing multiple hallmarks of this aggressive malignancy.

## 4 Materials and Methods

### 4.1 Processing and Integration of Single‐Cell RNA Sequencing Data

In this study, we employed two independent glioblastoma single‐cell RNA sequencing (scRNA‐seq) datasets derived from glioblastoma samples: GSE197543, which encompasses diverse cellular populations from both the tumour core and peripheral regions and GSE162631, consisting of CD31‐positive immune cells isolated from comparable anatomical locations. Initial data preprocessing involved the use of Seurat version 5, wherein quality control criteria excluded cells with fewer than 200 detected genes or exceeding 10% mitochondrial transcript content. Subsequent normalisation was performed utilising the SCTransform method. Dimensionality reduction was achieved via principal component analysis (PCA) retaining 30 principal components, followed by uniform manifold approximation and projection (UMAP) to facilitate data visualisation [29].

Cell type annotations from the GSE197543 dataset were transferred to the GSE162631 dataset using Seurat's label transfer functionality. To address batch effects and technical variation, the two datasets were integrated using canonical correlation analysis (CCA) with anchoring based on mutual nearest neighbours. The effectiveness of integration was assessed by examining the preservation of cell type clustering and the distribution of samples within the reduced dimensional space. Finally, comparative analysis of cell type proportions across sample groups was conducted using normalised cell count data.

### 4.2 Differential Gene Expression Analysis

NPC‐like tumour cells and CD8+ T lymphocytes were subsetted from the integrated dataset by subsetting cells annotated as ‘NPC‐like tumour cells’ and ‘CD8+ T cells’ according to the CellAnnotationFine classification schema. Differential gene expression analysis between central and peripheral regions was assessed using the FindMarkers function in Seurat, which applies the Wilcoxon rank‐sum test. The analysis parameters were set to a minimum expression percentage (min.pct0) of 0.1 and a log fold‐change threshold (logfc.threshold) of 0.25. Genes exhibiting adjusted *p*‐value below 0.05 were considered statistically significant. Visualisation of differentially expressed genes was accomplished through volcano plots generated with ggplot2 and ggrepel R packages, plotting log_2_ fold change on the x‐axis against the negative log10 of the adjusted *p*‐value on the *y*‐axis. Expression thresholds were defined as an absolute log_2_ fold change greater than 0.25 (corresponding approximately to a 1.2‐fold change) and an adjusted *p*‐value less than 0.05. Notably, genes of particular interest, including NUCB2, SCGN, IFI6 and COL5A2, were specifically annotated on the plots. Expression distributions were further illustrated using violin plots with expression percentages calculated as the proportion of cells expressing each gene within respective anatomical regions.

### 4.3 Pathway Analysis of CD8+ T Cells

Functional enrichment analyses were conducted employing the clusterProfiler R package [30, 31]. Gene Ontology (GO) enrichment was performed separately for the biological process (BP), molecular function (MF) and cellular component (CC) categories using the enrichGO function, incorporating Benjamini‐Hochberg correction for multiple hypotheses. KEGG pathway enrichment was assessed via the enrichKEGG function. For both GO and KEGG analyses, the background gene set comprised all genes detected within CD8+ T cells, and significance was defined by an adjusted *p*‐value threshold of less than 0.05. Additionally, Gene Set Enrichment Analysis (GSEA) was performed using gene lists ranked by log_2_ fold change against the Molecular Signatures Database (MSigDB) Hallmark gene sets (h_gene_sets) for *Homo sapiens* using the GSEA function from clusterProfiler. The GSEA was conducted with 10,000 permutations and applied Benjamini‐Hochberg adjustment to control for multiple testing. Enrichment plots for significant pathways meeting the significance criterion (adjusted *p* < 0.05) were generated using the gseaplot2 function, displaying normalised enrichment scores, gene ranks and running enrichment scores. Overall, pathways enrichment results were visualised through dotplots and barplots, highlighting GenRatio and adjusted *p*‐values as principal metrics.

### 4.4 Survival Analysis

Survival analyses were conducted utilising the TIMER 2.0 web platform (http://timer.cistrome.org/), incorporating gene expression and clinical data from The Cancer Genome Atlas (TCGA) GBM cohort (*n* = 153) as reported by Li et al. [32]. Patient samples were stratified into high and low expression groups based on the median expression level of each gene of interest. Kaplan–Meier survival curves were generated to visualise differences in overall survival, and hazard ratios (HR) with corresponding 95% confidence intervals were estimated using Cox proportional hazards regression models. Statistical significance was evaluated using log‐rank tests, with a significance threshold of *p* < 0.05. The Cox regression models were specified as Surv (OS, EVENT)~Gene, where OS denotes overall survival time in months and EVENT indicates mortality status. Model robustness was assessed through multiple performance metrics, including R‐squared values, likelihood ratio tests, Wald tests and score (log‐rank) tests.

### 4.5 Pan‐Cancer Expression Analysis

The expression profile of NUCB2 across various cancer types was investigated using TIMER 2.0 (Tumour Immune Estimation Resource, http://timer.cistrome.org/). RNA sequencing data derived from The Cancer Genome Atlas (TCGA) were employed to compare NUCB2 expression levels between tumour tissue and their corresponding adjacent normal counterparts across multiple cancer types. Data visualisation was conducted through box plots based on log_2_‐transformed transcripts per million (TPM+1) values. Statistical significance of differential expressions was evaluated using the Wilcoxon rank‐sum test.

### 4.6 Cell Culture and NUCB2 Knockdown

Human GBM cell lines U87MG and U251MG were procured from the American Type Culture Collection (ATCC) and cultured in Dulbecco's Modified Eagle Medium (DMEM) supplemented with 10% fetal bovine serum and 1% penicillin/streptomycin, maintained at 37°C in a humidified atmosphere containing 5% CO_2_. Authentication of cell lines was rigorously performed via short tandem repeat (STR) profiling prior to experimental use, confirming over 95% concordance with ATCC reference profiles for both U87MG and U251MG cell lines. Mycoplasma contamination was excluded through routine screening. For NUCB2 silencing, cells were transfected with shRNA plasmids targeting human NUCB2 or a non‐targeting control (shCTR) using Lipofectamine 3000 (Invitrogen) following the manufacturer's instructions. Knockdown efficiency was assessed 48 h post‐transfection, at which point subsequent functional assays were conducted. The sequences of the shRNAs were as follows: shNUCB2‐1, AAGCTGTGCCTATTGACATAGAC; shNUCB2‐2, AAGCAAAGAACTGGATTTAGTAA.

### 4.7 Quantitative PCR

Total RNA was isolated using TRIzol reagent (Invitrogen) and reverse transcribed employing the PrimeScript RT Reagent Kit (Takara). RNA integrity was verified by agarose gel electrophoresis, demonstrating distinct 28S and 18S ribosomal RNA bands with appropriate intensity ratios, indicating high‐quality, non‐degraded RNA. RNA concentration and purity were further determined spectrophotometrically, with A260/A280 ratios exceeding 1.8, ensuring suitability for downstream reverse transcription reactions. Only samples meeting these stringent quality criteria were subjected to complementary DNA (cDNA) synthesis. Quantitative polymerase chain reaction (qPCR) was performed using SYBR Green Master Mix (Applied Biosystems) on a QuantStudio 5 Real‐Time PCR System. Relative NUCB2 expression levels were normalised to GAPDH and calculated using the 2^−ΔΔCT^ method. All experiments were conducted in triplicate, with results presented as mean ± standard error of the mean (SEM).

### 4.8 EdU Incorporation Assay

Cell proliferation was assessed utilising the Click‐iT EdU Alexa Fluor 647 Imaging Kit (Invitrogen) in accordance with the manufacturer's instructions. Briefly, transfected cells (3 × 10^4^ per well) were plated onto coverslips within 24‐well plates on coverslips 24 h following transfection. After an additional 24 h of incubation, 10 μM of EdU was introduced into the culture medium for 2 h. Subsequently, cells were fixed with 4% paraformaldehyde, permeabilised using 0.5% Triton X‐100, and incubated with the Click‐iT reaction cocktail for 30 min. Nuclear counterstaining was performed with DAPI. Confocal images were acquired using a Zeiss LSM 800 confocal microscope equipped with a 20× objective, capturing five randomly selected fields per sample. Quantification of EdU‐positive cells was conducted by calculating the percentage relative to total DAPI‐positive nuclei using ImageJ software, with a minimum of 500 cells counted per condition across four independent biological replicates. Statistical comparisons were made using Student's *t*‐test, considering *p*‐values less than 0.05 as indicative of statistical significant.

### 4.9 Immune Cell Infiltration Analysis

The association between NUCB2 expression and immune cell infiltration in GBM was examined via TIMER 2.0, a computational platform that applies a deconvolution methodology to estimate immune cell infiltration levels from RNA sequencing data. Spearman's correlation coefficients, adjusted for tumour purity, were calculated to assess the relationships between NUCB2 expression and the infiltration levels of macrophages and CD8+ T lymphocytes.

### 4.10 Correlation Analysis With Immunoinhibitory Molecules

The potential correlations between NUCB2 expression and immunoinhibitory molecules were explored using the Tumour and Immune System Interaction Database (TISIDB, http://cis.hku.hk/TISIDB/), an integrated resource for tumour–immune system interactions [33]. Expression profiles from 166 GBM samples in The Cancer Genome Atlas (TCGA) were analysed to determine the correlations between NUCB2 and selected immunoinhibitory markers, including IL10RB, PDCD1LG2, IL10, IDO1 and CD96. Spearman's rank correlation coefficients (*ρ*) were employed to quantify correlation strength, with statistical significance defined as *p*‐value less than 0.05. Scatter plots incorporating linear regression lines were generated to visually represent these relationships.

## Author Contributions

**Xiaodong Huang:** data curation (equal), formal analysis (equal), investigation (equal), methodology (equal). **Xuemei Hu:** conceptualization (equal), formal analysis (equal), investigation (equal), methodology (equal). **Hang Xu:** data curation (supporting), formal analysis (supporting), investigation (supporting). **Bowen Yang:** data curation (supporting), formal analysis (supporting), investigation (supporting), methodology (supporting). **Lidong Yan:** data curation (supporting), formal analysis (supporting), investigation (supporting), methodology (supporting). **Junti Lu:** data curation (supporting), formal analysis (supporting), investigation (supporting), methodology (supporting). **Kuanming Huang:** data curation (supporting), formal analysis (supporting), investigation (supporting), methodology (supporting). **Huibin Chen:** resources (equal), supervision (equal), writing – original draft (equal), writing – review and editing (equal). **Shengli Hu:** resources (equal), supervision (equal), writing – original draft (equal), writing – review and editing (equal).

## Conflicts of Interest

The authors declare no conflicts of interest.

## Data Availability

The single‐cell RNA sequencing datasets analysed in this study are publicly available in the Gene Expression Omnibus (GEO) repository under accession numbers GSE197543 and GSE162631. Processed data, including differential gene expression results, pathway analysis outputs and computational scripts used for analyses, are available from the corresponding author upon reasonable request.
